# The ecology of suburban juvenile European hedgehogs (*Erinaceus europaeus*) in Denmark

**DOI:** 10.1002/ece3.5764

**Published:** 2019-10-31

**Authors:** Sophie L. Rasmussen, Thomas B. Berg, Torben Dabelsteen, Owen R. Jones

**Affiliations:** ^1^ Department of Biology University of Southern Denmark Odense M Denmark; ^2^ Behavioural Ecology Group, Ecology and Evolution Department of Biology University of Copenhagen Copenhagen Ø Denmark; ^3^ Naturama Svendborg Denmark; ^4^ Interdisciplinary Centre on Population Dynamics (CPop) Department of Biology University of Southern Denmark Odense M Denmark

**Keywords:** climate change, conservation ecology, *Erinaceus europaeus*, European hedgehog, urban habitats, wildlife ecology

## Abstract

1. European hedgehog (*Erinaceus europaeus*) populations are widespread across diverse habitats but are declining in Western Europe. Drastic declines have been described in the UK, with the most severe declines occurring in rural areas. Hedgehogs are widely distributed in Denmark, but their status remains unknown.
2. Fieldwork on hedgehogs has tended to focus on rural areas, leaving their ecology in suburban habitats largely unexplored, with clear implications for conservation initiatives. Here, we study the ecology of 35 juvenile hedgehogs using radio tracking during their first year of life in the suburbs of western Copenhagen.
3. We use radio‐tracking data to estimate (a) home range sizes in autumn and spring/summer, (b) survival during their first year of life, (c) the body mass changes before, during, and after hibernation, and (d) the hibernation behavior of the juvenile hedgehogs.
4. We show that males and females have small home ranges compared with previous studies. The 95% MCP home range sizes in autumn were 1.33 ha (95% CI = 0.88–2.00) for males and 1.40 ha (95% CI = 0.84–2.32) for females; for spring/summer they were 6.54 ha (95% CI = 3.76–11.38) for males and 1.51 ha (95% CI = 0.63–3.63) for females. The juvenile survival probabilities during the study period from September 2014 to July 2015 were .56 for females and .79 for males. All healthy individuals gained body mass during the autumn and survived hibernation with little body mass loss thus demonstrating that the juveniles in the study were capable of gaining sufficient weight in the wild to survive their first hibernation.
5. The climate is changing, but there is a lack of knowledge on how this affects mammal ecology. The exceptionally mild autumn of 2014 caused the juvenile hedgehogs to delay hibernation for up to a month compared with previous studies in Denmark.

## Graphical abstract

Juvenile European hedgehogs living in suburban habitats of Copenhagen, Denmark, were radio‐tagged and followed during their first year of life from September 2014 to July 2015. They had small home ranges, good survival rates, and gained weight efficiently. They delayed hibernation due to extremely mild climatic conditions.

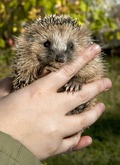

## 1 INTRODUCTION

There is substantial evidence that European hedgehog (*Erinaceus europaeus*) populations are declining based on monitoring data from the UK, Belgium, the Netherlands, Sweden, and Germany (Hof & Bright, 2016; Holsbeek, Rodts, & Muyldermans, 1999; Huijser & Bergers, 2000; Krange, 2015; Müller, 2018; van de Poel, Dekker, & Langevelde, 2015; SoBH, 2011, 2015, 2018; Williams et al., 2018). These declines are believed to be driven by habitat loss and fragmentation, intensified agricultural practices, road traffic accidents, molluscicide and rodenticide poisoning, and in some areas badger predation (Brakes & Smith, 2005; Dowding, Harris, Poulton, & Baker, 2010; Dowding, Shore, Worgan, Baker, & Harris, 2010; Haigh, O'Riordan, & Butler, 2012; Hof & Bright, 2010; Huijser & Bergers, 2000; SoBH, 2011; Young et al., 2006).

The study of hedgehog ecology in urban areas is underrepresented in the literature, even though hedgehogs seem to prefer residential areas (Doncaster, Rondinini, & Johnson, 2001; Hubert, Julliard, Biagianti, & Poulle, 2011; Pettett, Moorhouse, Johnson, & Macdonald, 2017; van de Poel et al., 2015). This preference could be due to higher food densities affiliated with human occupation, including natural prey and anthropogenic sources, more suitable nest sites and a decreased risk of predation by badgers (*Meles meles*; Micol, Doncaster, & Mackinlay, 1994; Morris, 1985; Pettett et al., 2017; Young et al., 2006). In the UK, it is furthermore suggested that the hedgehog decline is currently more severe in the rural than urban areas (SoBH, 2018; Williams et al., 2018). Since urban habitats may be more suitable for hedgehogs at the present time, it is relevant to describe the challenges hedgehogs face when living in this habitat type, to plan the optimal conservation initiatives directed at preserving hedgehogs in urban areas. The information gathered during a study on the ecology of hedgehogs in urban areas, for example, a lack of suitable nest sites, high mortality rates caused by dog attacks, and poisoning with rodenticides, can be applied to focus and optimize conservation efforts.

Previous studies on urban hedgehogs indicate that sheltered climatic conditions and anthropogenic food resources may be important predictors of increased presence of hedgehogs in urban areas compared with rural areas (Hubert et al., 2011). Dowding, Harris, et al. (2010) found that hedgehogs residing in urban areas primarily became active after midnight and avoided foraging near roads, likely to reduce the dangers and disturbances caused by human activities such as vehicle and foot traffic and the risk of predation by dogs (Morris & Reeve, 2008; Reeve & Huijser, 1999; Stocker, 2005). Green spaces in urban areas, such as parks, road verges, and gardens are often maintained thoroughly and may support several populations of wildlife, for example, amphibians and smaller mammals (Dickman, 1987). However, the fragmentation of the suitable habitats caused by roads, water‐bodies, and impenetrable fences is a challenge for the survival and genetic diversity of the populations (Braaker, Kormann, Bontadina, & Obrist, 2017; Hof & Bright, 2009). Hof and Bright (2009) suggested that initiatives taken by garden owners to increase the attractiveness of their gardens for wildlife, by adding features such as nest boxes and feeders, may attract hedgehogs to gardens. However, garden habitat quality in residential areas is weakened by the use of garden pesticides (insecticides, molluscicides, and rodenticides), which reduces the availability of natural food items for the hedgehogs and may cause secondary poisoning (Ditchkoff, Saalfeld, & Gibson, 2006; Dowding, Shore, et al., 2010).

### 1.1 Home range sizes of wild juvenile hedgehogs

A home range is the spatial area in which an animal concentrates its activities in a defined time period. It is a useful tool for understanding the spatial ecology of a species and for optimizing conservation initiatives, as animals focus their activities in their home range areas, because the spatial and temporal information about these areas, which are stored in the individuals' cognition, increases fitness (Spencer, 2012). Juvenile hedgehogs (<12 months of age) tend to have smaller home range sizes than adults (Kristiansson, 1984). When comparing measures of home range sizes, it is important to consider the methodology used, such as the number of observations leading to the calculations, the study duration, time frame, habitat type, monitoring method (capture‐mark‐recapture [CMR] or radio/GPS tracking) number of fixes per night, calculation methods such as minimum convex polygons (MCP) and kernel density estimates (KDE), and the life stage of the monitored individuals (Morris, 1988).

Using CMR, (Kristiansson, 1984) calculated home ranges of 9.2 ha (subadult males) and 3.4 ha (subadult females) in a Swedish village. Reeve (1982) found home range sizes of 10–15 ha during 83 nights of radio tracking (*n* = 3, aged 6–12 months) on a golf course in west London, UK. Sæther (1997) measured home range sizes of 2.6–3.0 ha (MCP) by radio tracking (*n* = 15, aged 4–9 weeks) for 4 weeks in a residential area near Trondheim, Norway. Furthermore, Kristiansson and Erlinge (1977) reported a home range size of 3.7 ha (*n* = 1, aged 3 months, October, Sweden) and Rasmussen (2013) found home ranges of 3.54 and 4.85 ha (MCP; *n* = 2, aged 3–4 months, October–November, Denmark). In comparison, 10 adult hedgehogs were radio tracked in a rural area near Århus, Denmark, in the summer of 2005, generating mean MCP home range sizes of 96 ± 24 ha (mean ± *SD*, males, *n* = 4) and 26 ± 15 ha (mean ± *SD*, females, *n* = 4; Riber, 2006). Only the two studies from Denmark (Rasmussen, 2013; Riber, 2006) used GPS as a tool for registering the positions of the radio tracked hedgehogs. In conclusion, previously recorded home range sizes of wild juvenile (up to 12 months of age) hedgehogs range between 2.6 and 15 ha.

### 1.2 Survival probabilities of wild juvenile hedgehogs in Scandinavia

The viability of a population is heavily influenced by the survival probability of the individuals in the population. A high survival probability of juvenile hedgehogs during their first year of life is important for the growth of population because surviving juveniles will eventually join the breeding population. Reported survival probabilities of Scandinavian juvenile hedgehogs have varied widely depending on age, background (rehabilitated or wild), the time of year, habitat type, tracking method, location, and climatic conditions in the study period (Jensen, 2004; Kristiansson, 1984, 1990; Rasmussen, 2013; Sæther, 1997; Walhovd, 1990). Sæther (1997) found an autumn survival probability of .31 for 25 radio‐tagged juvenile hedgehogs (during their 4th–9th week of life) in a residential area in Norway. Kristiansson (1990) estimated an average annual juvenile survival of .66 (*n* = 123), using CMR in a Swedish village. A prehibernation survival probability of .50 was estimated for 10 wild, radio‐tagged independent juveniles in a recreational area near Copenhagen, Denmark (Rasmussen, 2013), while Jensen (2004) found a 100% winter survival for seven Danish radio‐tagged juvenile hedgehogs. In summary, survival probabilities for Scandinavian hedgehogs range between .31 and 1.00 depending on the age and period of time in which they were studied.

### 1.3 Hibernation of juvenile hedgehogs in Scandinavia

Surviving their first hibernation is a challenge for juvenile hedgehogs. Survival is dependent on factors such as nest quality (Morris, 1973), health (particularly the amount of fat deposits, Kristiansson, 1990), and temperature during the hibernation period (Morris, 2018). The optimal prehibernation body mass for juveniles to survive hibernation has been debated for years. Morris (1984) suggested a minimum body mass of 450 g in order to survive hibernation, and studies on Danish juveniles also indicated that ≥450 g would be sufficient (Jensen, 2004; Rasmussen, 2013).

Considering past research on hibernation body mass and survival of juvenile hedgehogs in Scandinavia, Jensen (2004) found that six Danish rural‐living juvenile hedgehogs all survived (513–897 g prehibernation), using two–four nests. Walhovd (1990) recorded an average spring recapture rate (CMR) of 69% among juvenile hedgehogs (400–800 g prehibernation) in Denmark, during 6 years of study. Likewise, Kristiansson (1984) estimated the winter survival in southern Sweden to be an average of 67% for all age categories, based on CMR.

In summary, past studies on the hibernation of juvenile hedgehogs in Scandinavia found that the onset of hibernation is normally between late October and November, and activity is resumed from mid‐April to mid‐May that juvenile hedgehogs change nests 0–4 times during winter and the winter survival ranges between 60% and 100% (Jensen, 2004; Kristiansson, 1984, 1990; Rasmussen, 2013; Walhovd, 1976, 1978, 1990).

### 1.4 The potential effects of climate change on hedgehogs

Anthropogenic climate change has already impacted wild species (Parmesan et al., 2013), shifting their geographic ranges and seasonal activities (Intergovernmental Panel on Climate Change, 2014). Understanding how climate change may affect hedgehog ecology is therefore essential for the conservation of the species. Because weather conditions are believed to be one of the possible triggers for the onsets of the breeding season and the hibernation for hedgehogs (Morris, 2018), it is likely that changes in weather patterns driven by climate change will influence their survival and reproductive output.

In hedgehogs, warmer winters could pose a particular risk, because the periodic rises in temperature (to ≥10°C) may induce arousals from torpor (Kristoffersson & Soivio, 1964; Newman & Macdonald, 2015), initiating the thermoregulatory responses that lead to increased metabolic rates and a rapid return of body temperature to the normal levels (Carey, Andrews, & Martin, 2003), which may increase the drain of fat reserves of the hedgehogs. Furthermore, drier seasons in general could limit the amount of food items such as earthworms, since their abundance and distributions are sensitive to microclimatic conditions such as soil moisture (Edwards & Bohlen, 1996; Macdonald, Newman, Buesching, & Nouvellet, 2010). The warmer and wetter conditions may also cause increasing viability, population sizes, and biting rates of a range of disease vectors infecting hedgehogs such as ticks, potentially carrying Lyme disease (Gern, Rouvinez, Toutoungi, & Godfroid, 1997; Harvell et al., 2002; Macdonald, Moorhouse, & Gelling, 2014).

So far, research on the effects of climate change on hedgehog ecology remain sparse. A study on the effect of climate change on posthibernation emergence of hedgehogs in the UK showed that the emergence timings of hedgehogs do appear to be linked to variation in local climatic conditions (PTES & BHPS, 2015). However, a general effect of climate change could not be found.

### 1.5 The status of the Danish hedgehog population and the aim of the research

Due to the lack of monitoring of hedgehogs in Denmark, their conservation status remains unknown. It is however likely that the decline found in other European countries is similar in Denmark, which has comparable habitat fragmentation, landscape structure, farm management practices, and climate to other countries with a detected decline. Hopefully, the current ongoing research on the Danish hedgehog population will eventually enable an estimation of their conservation status.

The aim of this study is to describe the ecology of juvenile hedgehogs residing in suburban habitats during their first year of life. Specifically, we fill a knowledge gap that exists on suburban‐living juvenile hedgehogs by reporting home range size estimates, survival, body mass change, and hibernation behavior during their first year of life from September 2014 to July 2015. Obtaining these data will improve our understanding of the challenges juvenile hedgehogs face and how these affect their survival.

## 2 MATERIALS AND METHODS

### 2.1 Home range size

To estimate home range size we radio‐tagged and tracked 35 independent juvenile hedgehogs (14 females and 21 males) in the western suburbs of Copenhagen, Denmark from 20 September, 2014 to 22 July, 2015. These areas are dominated by housing and private gardens, and we obtained access permission via a local media advertising campaign. Most hedgehogs were caught by manually searching through public hedgehog‐friendly areas with torches or headlights. To increase trapping success in private gardens, we placed a Bolyguard MMS 550M 8 MP wildlife camera in front of feeding stations situated in gardens, where juvenile hedgehogs were known to appear. The camera sent a picture to the smartphone of the researcher waiting outside the garden whenever movement was detected at the feeding station, enabling efficient capture while minimizing disturbance. We attached the radio tags (PIP or TW3 tags from Biotrack Ltd, weighing 3 and 11 g, respectively) to the hedgehog's spines using quick‐drying two‐part epoxy glue and, to aid recovery, we added reflective tape to the tags. Our radio transmitters weighed <5% of the hedgehogs' body mass, in accordance with the guidelines of the American Society of Mammalogists (Sikes, 2016). We released the animals after a clinical examination and weighing on a digital kitchen scale with a built‐in bowl (OBH Nordica Mix&Weigh 9835) which we sanitised with alcohol between uses. We tagged most animals (33) between 21 September and 26 November, 2014, and a further two posthibernation in April 2015. A small number (*n* = 3) of the initial 33 tagged were rehabilitated, orphaned siblings. All tagged animals were independent from their mothers (>6 weeks of age) when entering the study and weighed between 213 and 659 g when tagged.

During the autumn, spring, and summer of 2014–2015, we radio tracked these hedgehogs using a Sika receiver and Yagi antenna during their activity periods between sunset and sunrise. This work represented 70 nights of fieldwork in the autumn of 2014 and 84 nights of fieldwork during the spring and summer of 2015. Each night we made position estimates for the animals with 1‐hr interval, and we recorded the positions using Garmin Dakota 20, Garmin eTrex 20, and Garmin Oregon 200 GPS devices. Due to the geographical dispersal of the individuals, each animal was radio tracked approximately one night a week. If the animal was located inside an inaccessible garden, we recorded the location as being on the pavement just outside the garden. We continued radio tracking once a week during the hibernation period and also recorded the number of nest changes during this period. Additionally, we monitored the weather conditions during the study period by extracting data from the Danish Meteorological Institute to investigate the effects of local climatic conditions on the behavior of the hedgehogs, especially the timing of hibernation.

**Figure 1:**
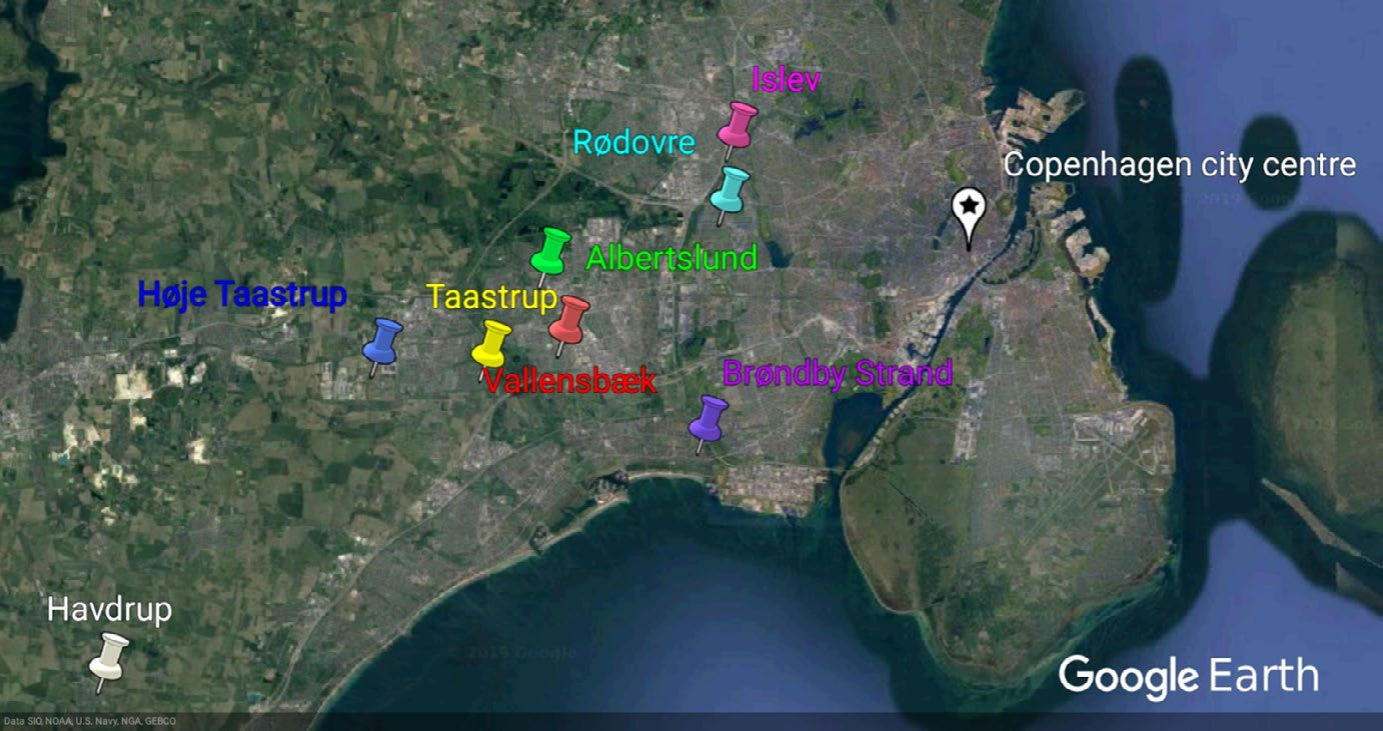
A map of Zealand, Denmark showing the locations of the radio tracked hedgehogs. Seven western suburbs were chosen (Islev, Rødovre, Albertslund, Taastrup, Høje Taastrup, Brøndby Strand and Vallensbæk), as well as the provincial town of Havdrup

We radio tracked juvenile hedgehogs in seven western suburbs of Copenhagen and the more provincial town of Havdrup (Figure 1 and Table 1).

**Table 1:** Overview of study sites

Location	GPS location	Area (km^2^)	Citizens	Population density (inhabitants/km^2^)	Roads (km)	Hedgehogs tagged
Albertslund	55.664356, 12.350708	23.04	27,877	1,210	8,749	1
Brøndby Strand (Brøndby municipality)	55.622937, 12.418683	20.85	35,219	1,689	14,092	2
Havdrup (Solrød municipality)	55.535898, 12.119176	12.75	4,302	337	14,512	5
Rødovre/Islev	55.677220, 12.452584/55.697346, 12.453308	12.8	40,052	3,129	4,616	10 (9/1)
Taastrup/Høje Taastrup	55.640174, 12.329802/55.647205, 12.264672	78.32	50,686	647	20,350	16 (11/5)
Vallensbæk	55.631698, 12.366053	9.23	16,654	1,804	4,804	1

Note

Information on the eight radio tracking locations used in the study. Taastrup/Høje Taastrup and Rødovre/Islev have been merged in the table because they belong to the same municipality. The measures for Havdrup and Brøndby Strand are based on the data from the entire municipalities of which they belong. The data for this table are collected from (Statistics Denmark, 2019a, 2019b; Vejdirektoratet, 2019).

We estimated home range sizes for individuals for which we had at least 30 location points both before and after hibernation (Seaman et al., 1999). To do this, we used the package adehabitatHR version 0.4.16 (Calenge, 2006) in R (R Core Team, 2019) to calculate the 50% and 95% minimum convex polygons (MCP) and 50% and 95% kernel density estimates (KDE) for each individual in both autumn and spring/summer (*h* = LSCV).

To investigate whether home range size varied between seasons and sexes, we fitted generalized linear models (GLMs) in R (R Core Team, 2019) with a Gamma error structure and log link, to account for non‐normality. Our response variable was home range size in hectares, and the explanatory variables were sex (female/male) and season (autumn/spring). We first fitted a maximal model including both explanatory variables and the two‐way interaction between them (sex, season, sex:season). We checked whether the models could be simplified by removing nonsignificant terms, for example, the interaction term (Crawley, 2013) by examining the analysis of deviance table for each model.

### 2.2 Mortality and cause of death

In addition to tracking position, we collected data on mortality events in the hedgehogs we were following. If the cause of death was unclear, the hedgehogs were necropsied at http://www.Wildlifehealth.dk to clarify this information. Based on the mortality data, we calculated Kaplan–Meier survival rates for all individuals combined and for both sexes, respectively. We used the log‐rank (Mantel‐Cox) test to test for differences between the survival rates.

### 2.3 Body mass and nutritional status

To monitor the health and development of the juvenile hedgehogs, we registered their nutritional status and body mass. After the initial weighing and radio tagging, we weighed the hedgehogs as close to the onset of hibernation as possible. For posthibernation body mass, the hedgehogs were caught and weighed at the first opportunity. Some hedgehogs were furthermore weighed during the autumn and spring. Due to challenges predicting the hibernation onset for each individual, and thereby obtaining a body mass measure just before the onset of hibernation, we created linear regressions based on body mass change during autumn for individuals with ≥2 recorded body mass measures, to estimate the body mass of the hedgehogs on the exact dates of hibernation onset. These measures are hereafter referred to as the “estimated body mass.”

We used the Bunnell Index (BI; Bunnell, 2002) as a measure of nutritional status. This index is calculated as the ratio of the circumference of the curled‐up hedgehogs crosswise (A) and lengthwise (B) (i.e., A divided by B), by using a soft retractable measuring tape. A BI of >0.8 is associated with a healthy animal with a satisfactory body mass/size ratio.

### 2.4 Hibernation and nest changes

We radio tracked the hedgehogs once a week during hibernation, registering the nest changes and the locations and types of nests used during hibernation (e.g., “under garden shed,” “inside compost heap”). The radiotracking was intensified around mid‐April, from when the hedgehogs were expected to become active.

### 2.5 Protection of animals in research

Our research was carried out in accordance with Danish Law (The Administrative Order on the Protection of Species, Artsfredningsbekendtgørelsen) and a permit to radio tag hedgehogs was granted by the Danish Nature Agency in September 2014 (J. Nr. SNS‐41500‐00210).

## 3 RESULTS

### 3.1 Home range size

**Figure 2:**
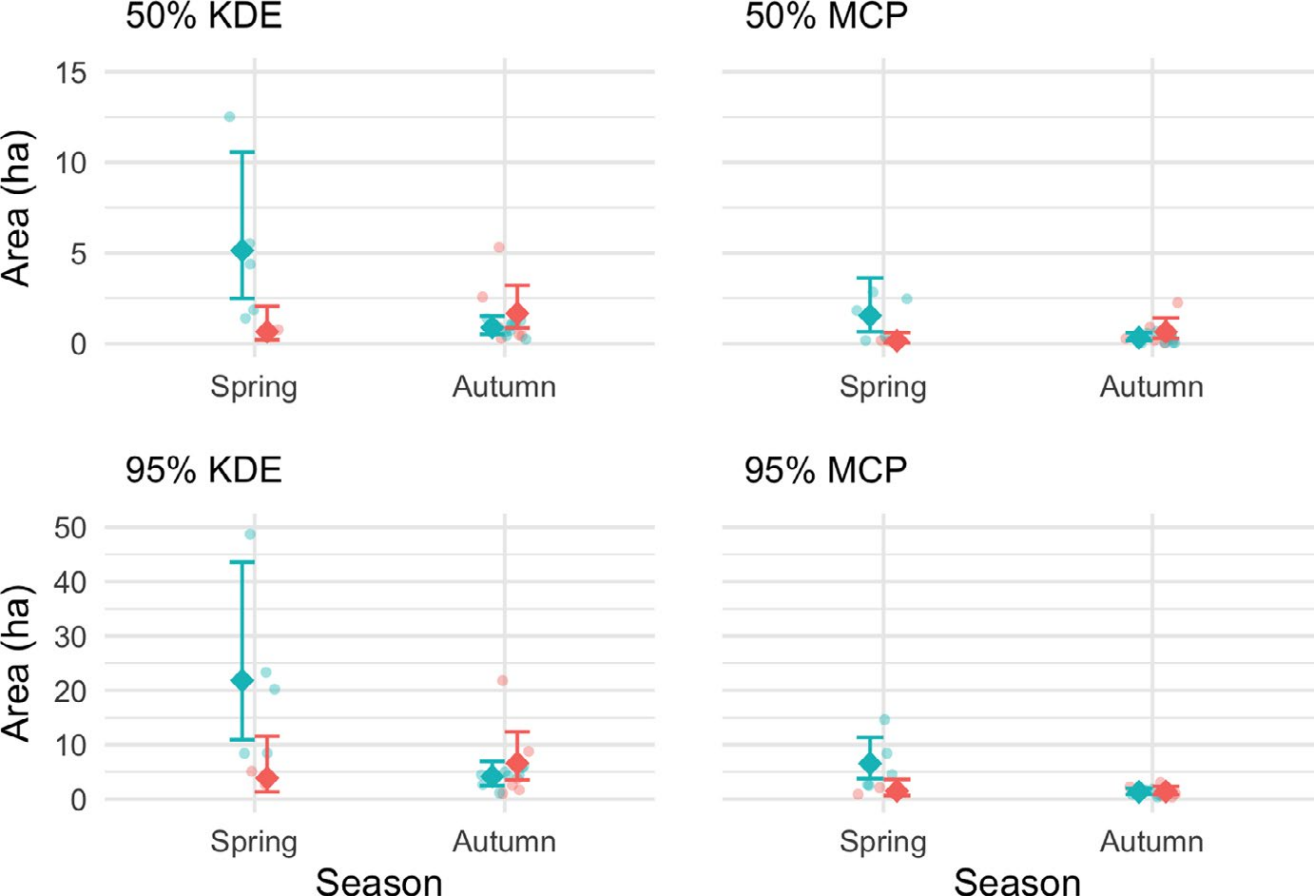
Home range estimates during spring/summer (2015) and autumn (2014) for 22 male and female hedgehogs for individuals with at least 30 recorded GPS locations. Each subplot represents the results obtained using one of four home range estimation methods (50% MCP, 95% MCP, 50% KDE, and 95% KDE). The pale points represent individual estimates of home range size (jittered), the darker points with error bars represent the fitted values, and 95% confidence intervals estimated from the GLMs. Blue points represent males and red points represent females

It was possible to calculate 22 home range areas using 95% and 50% minimum convex polygons (MCP) and 95% and 50% kernel density estimates (KDE) based on ≥30 GPS coordinates per individual during the autumn of 2014 and/or the spring/summer of 2015 (Table 2, Figure 2 and Appendix S1). The GLMs for home range size showed that, for all four methods, home range size was associated with sex and season, and that there was an interaction between these two variables. This interaction was statistically significant in all cases: KDE50: *χ* ^2^ = 5.547, *df* = 1, *p* = .003; KDE95: *χ* ^2^ = 3.950, *df* = 1, *p* = .010; MCP50: *χ* ^2^ = 6.529, *df* = 1, *p* = .007; MCP95: *χ* ^2^ = 2.051, *df* = 1, *p* = .020. The interaction indicates that the effect of sex depends on the season and this is shown clearly in Figure 2: Males tend to have larger home ranges than females, but only during the spring. This effect is qualitatively similar with all home range estimation methods. Females tend to have slightly larger mean home range sizes than males in autumn, though examination of the confidence intervals shows that this effect is not statistically significant.

**Table 2:** Mean home range sizes

Season	Sex	KDE50	KDE95	MCP50	MCP95
Autumn	Male	0.89 (0.52–1.52)	4.15 (2.48–6.95)	0.32 (0.17–0.61)	1.33 (0.88–2.00)
Autumn	Female	1.67 (0.86–3.21)	6.60 (3.51–12.41)	0.65 (0.30–1.41)	1.40 (0.84–2.32)
Spring	Male	5.14 (2.50–10.56)	21.83 (10.93–43.59)	1.54 (0.66–3.61)	6.54 (3.76–11.38)
Spring	Female	0.66 (0.21–2.06)	3.88 (1.30–11.57)	0.16 (0.04–0.61)	1.51 (0.63–3.63)

Note

Mean home range sizes vary with season and sex, and depending on the method used. The methods used were 50% and 95% kernel density estimates (KDE) and minimum convex polygons (MCP). Values are given in hectares, with the 95% confidence intervals in parentheses.

The young males in Taastrup (Figure 1) expanded their home ranges during the mating season to include novel areas. Individuals visited on average 10 gardens (mean garden size: 0.95 ha or 950 m^2^, range = 0.045–0.125 ha) during the autumn of 2014 (range = 2–20, *n* = 22), though not necessarily each night. This average rose to 14 gardens during spring/summer of 2015 (range = 7–20, *n* = 10).

The two most distant location points recorded per individual during the autumn ranged between 79 and 908 m, with a mean of 285 ± 197.7 m (mean ± *SD*, *n* = 22). The distance ranged between 156 and 843 m in the spring/summer, with a mean of 388 ± 206.4 m (mean ± *SD*, *n* = 10; see Appendix S1).

The radio signals were lost from seven individuals during the study out of which two individuals were unaccounted for just after their tagging, perhaps due to defective tags. The seven individuals could not be found again, and the signals from the radio tags were never retrieved in spite of a thorough search effort within a radius of 5 km from where they were last spotted. Due to the shedding of juvenile spines into adult spines, six individuals lost their tags before hibernation, two during hibernation, and four after hibernation (Table 3 and Appendix S1). Fortunately, we occasionally managed to reattach the tags to the same individuals again, leaving the total loss of tags to twelve incidences. One individual had two radio tags reapplied in the months after hibernation using a total of three tags in the study period. Several individuals had their glue reinforced or the tag moved and reattached whenever it was apparent that the tag became more and more detached and flabby due to the gradual shedding of spines. When retrieved, the detached tags were in good condition, with all the glue intact and a large number of spines attached to the glue. Due to the loss of tags before and during hibernation, eight individuals were excluded from the hibernation study. One individual was however seen alive in July 2015 and therefore counts as a survivor in the study. Four individuals lost their tags after hibernation. These individuals were never caught and radio‐tagged again in spite of a thorough search effort.

**Table 3:** Overview of individuals in the study

	Before hibernation	During hibernation	After hibernation
Individuals tagged	32	0	2
Signals lost	3	0	4
Tags lost	6	2	4
Individuals dying	6	2	1
Individuals surviving	18/23	16/18	7/8
Percentage survival	78	89	88

Note

An overview of the number of dead individuals, the number of lost signals, and tags (the individuals unaccounted for) before, during, and after hibernation. The survival rates are only based on individuals that could be accounted for in the study (e.g., 18 out of 23).

### 3.2 Mortality and cause of death

Nine of the 35 tagged individuals died during the study, of which two died during hibernation. Altogether, 18 out of 23 survived with certainty until the onset of hibernation. Two individuals died during hibernation. Seven individuals were known to have survived from their awakening from hibernation in late April or mid‐May until the end of July. Unfortunately, 12 individuals lost their tags (six before hibernation, two during hibernation, and four after hibernation) and seven individuals were unaccounted for due to lost radio signals (three before hibernation and four after hibernation; Table 3).

**Figure 3:**
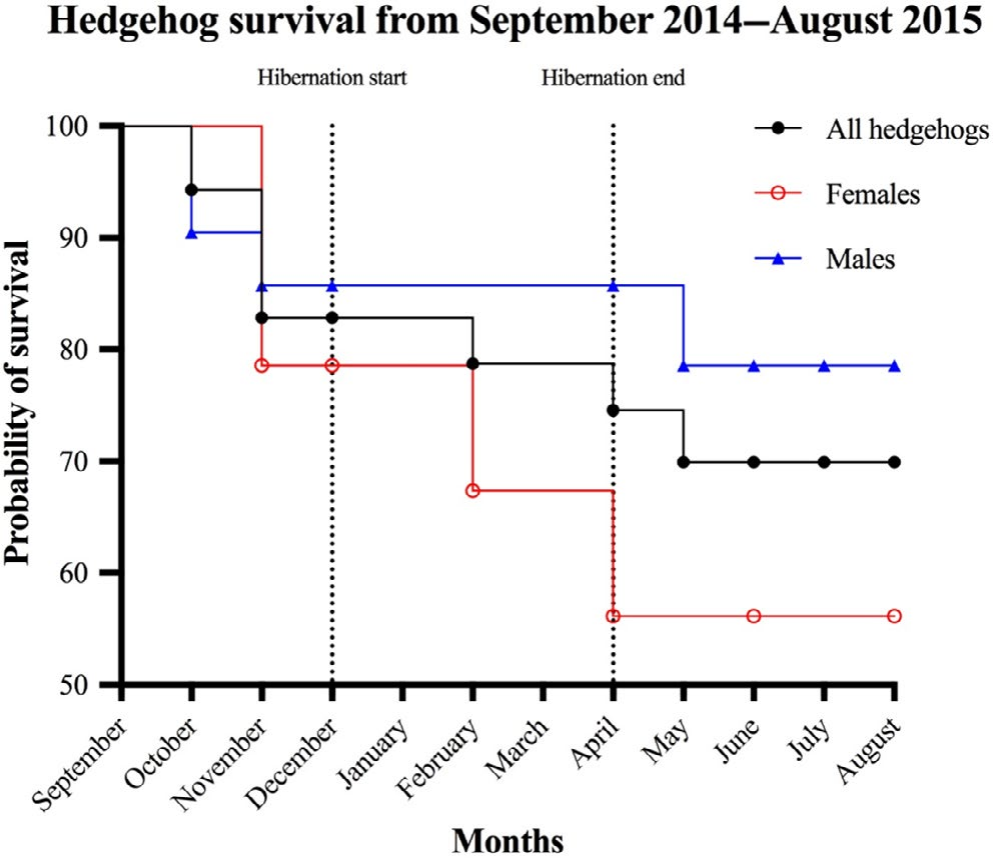
Kaplan–Meier survival curve representing the total number of juvenile hedgehogs (*n* = 35, black) in the study as well as the females (*n* = 14, red) and males (*n* = 21, blue). Twenty‐six data points were censored, due to unknown fate after the study ended (*n* = 7), loss of radio tag (*n* = 12), and loss of radio signal (*n* = 7). The calculated survival proportions with censored data were 70% for all hedgehogs, 56% for females, and 79% for males. The log‐rank (Mantel‐Cox) test showed no significant difference between the survival curves (*χ* ^2^ = 1.286, *df* = 2, *p* = .5257)

The Kaplan–Meier survival probabilities for the period of September 2014–July 2015 were .70 for all individuals combined and .56 for females and .79 for males, with 26 cases of censored data (12 lost tags, seven lost signals, and seven individuals surviving until the end of the study, Figure 3). The log‐rank (Mantel‐Cox) test showed no significant difference between the survival curves (Chi square = 1.286, *df* = 2, *p* = .5257).

The causes of death in these individuals varied. One individual was euthanized at Copenhagen Animal Hospital due to a self‐inflicted, lethal injury, as it was entangled in thorn branches, and another was taken into care by a hedgehog rehabilitator but did not survive. Necropsies of two individuals, performed at Wildlifehealth.dk, revealed that they died from *Salmonella* infections: one during the autumn and one during hibernation. Both visited the same feeding station, from where they likely contracted the infection. Green, watery, and smelly feces was observed near the food bowl visited by several hedgehogs including the two radio‐tagged individuals during the autumn of 2014. One individual drowned in an artificial stream surrounded by high concrete walls, and another may have died from poisoning with rodenticides but was not tested. Two individuals died due to fox attacks (one during hibernation) and one due to either a dog or fox attack. The last cause of death was shredding with garden waste, as the day nest was situated inside a pile of branches, which was destroyed in a garden waste shredder.

### 3.3 Body mass and nutritional status

All individuals gained body mass during autumn, except for two individuals dying from *Salmonella* infections. Healthy individuals weighing from 213 g in September reached a body mass of up to 755 g prior to hibernation (Table 4).

**Table 4:** Body mass change before and during hibernation

Individual	Body mass before hibernation				Body mass after hibernation						
	Hibernation start	Date of weighing	Body mass (g)	Calculated body mass at hibernation onset (g)	Hibernation end (2015)	Date of weighing (2015)	Body mass (g)	Body mass change (g)	Calculated body mass change (g)	% Body mass change	% Calculated body mass change
1	19/11/14	2/11/14	505	624							
2	21/11/14	29/10/14	570	801	2/5/15	13/5/15	653	83	−148	15	−18
7	16/11/14	29/10/14	755	924	24/4/15	14/5/15	665	−90	−259	−12	−28
8	19/11/14	1/11/14	748	947	24/4/15	24/4/15	577	−171	−370	−23	−39
14	11/11/14	16/11/14	504								
16	28/11/14	15/11/14	551	676	29/4/15						
17	5/12/14	20/10/14	470		9/5/15	19/5/15	754	284		60	
18	30/11/14	16/11/14	480	574	2/5/15	19/5/15	645	165	71	34	12
20	21/11/14	11/11/14	586	657	29/4/15						
23	30/11/14	15/10/14	424		14/4/15						
25	30/11/14	22/11/14	525	574	14/4/15	21/4/15	483	−42	−91	−8	−16
27		30/10/14	373								
28	2/1/15	6/1/15	472	379	14/5/15	14/5/15	446	−26	67	−6	18
29	5/12/14	29/10/14	488		24/4/15	19/5/15	752	264		54	
30	2/12/14	11/11/14	553								
31	8/12/14	17/11/14	656	807	30/4/15	17/5/15	749	93	−58	14	−7
32	8/12/14	2/11/14	589	1,148	30/4/15	30/4/15	590	1	−558	0	−49

Note

The hibernation and body mass data of the hedgehogs in the study. Individuals one and 14 died during hibernation. Individual no. 23 was killed a few days after hibernation and was therefore not weighed after hibernation. Individuals 16, 20, 27, and 30 lost their tags during hibernation or when waking up and leaving the nest, which made it impossible to obtain their posthibernation body mass. Individual 28 hibernated in a nest box at a hedgehog rehabilitation center (0 nest changes). The body mass provided in column 4 represents the last weighing before hibernation onset. The calculated body mass is based on linear regression (for individuals with ≥2 body mass records during autumn) in order to provide an estimate for the body mass on the exact date of hibernation onset.

Some individuals took a large effort to catch both before and after hibernation, causing their before and after body mass measures to be somewhat unrepresentative of the actual body mass loss during hibernation (e.g., individuals 17 and 29, Table 4). However, we measured the body mass change during hibernation with the available data and estimated the body mass change based on the exact date of hibernation by use of regression lines (Table 4).

Of the 10 individuals with sufficient data to represent the change in body mass during hibernation, four lost body mass, five gained body mass, and one had no body mass change (mean = +13% ± 2.8 *SE*). It was possible to calculate the estimated change in body mass from the date of hibernation onset for eight individuals, out of which six lost body mass and two gained body mass (mean = −16% ± 2.9 *SE*). The individuals losing body mass during hibernation, for example, 12% body mass loss from 755 to 665 g (estimated body mass loss of 28%), were in very good condition before hibernation. One individual lost 23% body mass during hibernation (estimated body mass loss of 39%), which was the highest recorded body mass loss of this study. This individual made eight nest changes during the hibernation period, and perhaps even more unrecorded, and was active almost weekly throughout the winter. The individual was still in good condition after awakening from hibernation, weighing 23% less (577 g). Very few individuals, only the two described earlier, had a documented body mass loss of more than 50 g during hibernation. The autumn and winter weather during this study was exceptionally mild (Danish Meteorological Institute, 2014, 2015b), leaving the hedgehogs in excellent condition before hibernation and hence in good condition after hibernating.

The female hedgehogs gained body mass throughout the spring/summer. One male kept a relatively steady body mass in spite of a large expansion of home range during the mating season. However, two males weighed from mid‐May and onwards lost body mass during the mating season.

Nutritional status, as estimated by the Bunnell Index (BI), varied markedly among animals with some being fairly healthy and others emaciated. We calculated 36 BIs for a total of 21 individuals, and the overall mean BI was 0.82 (range 0.71–0.90). One individual had a BI of 0.74 when found in a severely emaciated state, dying from *Salmonella* infections on the 2nd of November 2014. However, the individual with the lowest BI of 0.71, weighing 748 g on the 19th of May 2015 was in excellent condition (See Appendix S1).

### 3.4 Hibernation and nest changes

In 2014, the first night frost was on November 30th (Danish Meteorological Institute, 2014) and we recorded the first individual (out of 16) to enter hibernation on November 16th weighing >700 g. Six individuals began hibernation between November 16th and 21st, four individuals went into hibernation between November 28th and 30th and five individuals waited until December 2nd–8th. The last tagged individual began hibernation on 2 January, 2015 at a wildlife rehabilitation center.

The number of nest changes recorded per individual (*n* = 15) during hibernation ranged between 0 and 8, with most individuals changing nest 0–1 times (median = 0, range = 0–8, mean = 1.2 ± 0.14 *SE*). Most individuals stayed in one garden during hibernation but others ranged more widely: the individual with eight recorded nest changes visited at least 10 gardens during the hibernation period. The animals resumed posthibernation activity between 4 April and 14 May, 2015 (*n* = 13). The nest types used are described in Appendix S1.

## 4 DISCUSSION

Our results showed relatively small home range sizes and a high survival probability of radio tracked individuals living in a suburban habitat, compared with previous studies of juvenile hedgehogs. In the following section, we will compare the results to past research and discuss the application of the Bunnell Index as a measure of hedgehog health, as well as the effects of local climatic conditions on the ecology of the hedgehogs. We will furthermore consider the challenges of radio tagging juvenile hedgehogs and radio tracking in a suburban habitat and provide suggestions for improvements of this type of research in the future.

### 4.1 Home range size

The home ranges calculated in this study (Table 2) were generally small in comparison with home ranges from previous studies from Scandinavia, which have reported home range sizes of 2.6–9.2 ha which were however measured with different methods (Kristiansson, 1984; Rasmussen, 2013; Riber, 2006; Sæther, 1997). This could indicate that Copenhagen's suburban environment provides sufficient food resources for the hedgehogs enabling them to save energy on foraging and keeping smaller home ranges. We also observed that individuals tended to stay in the vicinity of the local feeding station with cat food and water, moving around in gardens surrounding this particular garden, especially during the autumn of 2014, as was the case with an individual only residing in approximately 4–5 gardens during the autumn. We did not regard the feeding stations as confounding factors in our study, since making feeding stations for hedgehogs is popular in suburban areas and the presence of feeding stations should therefore be regarded as normal conditions for suburban hedgehogs (Morris, 1985).

We found a statistically significant interactive effect of sex and season on the home ranges sizes, with home range sizes of males being larger than those of females during the spring/summer and similar during the autumn. The small trend for the home range sizes of females being larger than those of males in the autumn was nonsignificant, but this could be due to the small sample size for this comparison, for example, the effect of one particularly active female during the autumn of 2014. Our results are largely consistent with earlier studies which showed that male hedgehogs tend to have larger home ranges than females, and this perception is generally accepted (Morris, 2014). Our result contrasts with those of Sæther (1997) who found no difference between the home ranges of male and female juveniles living in a residential area near Trondheim, Norway, during the summer of 1995.

Few of the hedgehogs in our study dispersed more than 500 m from where they were initially radio‐tagged during the 10 months of the study, even though many of the individuals were caught in their birth area and were expected to disperse (Berthoud, 1978; Morris, 2014; Reeve, 1994). This surprising result could be explained by Doncaster et al. (2001) who suggested, that hedgehogs do not have a fixed natal territory from which to disperse, nor a clearly defined dispersal stage. This is supported by Sæther (1997) who found that newly independent male juveniles dispersed only 230 m from the natal nest and females dispersed 223.5 m from the natal nest.

During the present study, we observed one incidence of parents chasing their own juvenile offspring away from the feeding station in their natal area after hibernation, documenting that the juveniles never dispersed after reaching independence. However, these particular parents did show a range of deviant behavior, as they accepted the presence of their independent offspring during the autumn and they stayed in the same area and remained in a sort of cohabitation after the mating season, even though hedgehogs are solitary and males do not take part in the rearing of the young (Morris, 2018). This particular male and female both lived in the same garden shed from September 2014 to June 2015. The male slept in a nest next to the nest inhabited by the female and her offspring. The adults would follow the juveniles around together from the time the juveniles would leave the nest (at 3–4 weeks of age) until they reached independence (at around 6–7 weeks of age).

A large number of tags (12 out of 35 attached) were lost possibly due to the shedding of spines. This is a high number compared with previous studies using the same tag types, glue and attachment techniques. The shedding of spines, as they are replaced by adult spines, is normally a gradual process, but perhaps the excellent food availability caused by the great climatic conditions in the autumn, made the hedgehogs develop their adult spines faster making the shedding more sudden than the normal gradual process? Perhaps the hedgehogs in the study could have become stuck under garden fences more often compared with individuals previously studied in more open landscapes with less of those features. Most shed tags were retrieved and had intact glue with several spines still attached. The spines had bulb roots at the end as seen in regular shedding processes. The loss of radio tags due to shedding reduced the number of individuals followed throughout the study, until the last night of fieldwork in July, to seven, regrettably limiting the sample size of calculated home ranges.

The loss of signals from the radio tags could be explained by hedgehogs falling victims to traffic, since the collision may cause the tags to break and scavengers may have eaten or removed the roadkills (and radio tags) from the area. Apparently, no individuals were hit by cars during the study, but at least two with lost signals were known to cross relatively busy roads in the summer, where a number of hedgehogs are killed by cars every year. These roads are frequently visited by scavengers like foxes and gulls effectively removing roadkills. A signal was lost from an individual in the more rurally situated suburb of Havdrup, the only study site inhabited by badgers, which could be a plausible explanation for the disappearance of this particular individual. Foxes were present at all the locations.

We observed that the spatial activity of individuals, in particular from Taastrup and Rødovre, appeared to be limited by the major roads in the area. Individuals tended to avoid crossing the major roads and would focus their activities in areas on either one or the other side of the road, often along the axes formed by the road. This behavior could be interpreted as a clear indication of habitat fragmentation.

### 4.2 Survival

A high survival probability of juvenile hedgehogs during their first year of life is important for the growth and maintenance of the general hedgehog population. The survival probabilities for the hedgehogs in this study (Kaplan–Meier: .7 for all individuals, .56 for females and .79 for males) were rather high compared with previous studies of juvenile hedgehogs in Scandinavia, which range between .31 and .66 (Kristiansson, 1990; Rasmussen, 2013; Sæther, 1997). The survival probability of .89 (16 out of 18 individuals) during hibernation in this study is also quite high compared with the estimates of .66 and .69 by (Kristiansson, 1984) and Walhovd (1990). Nevertheless, Jensen (2004) found a 100% survival rate during hibernation for the six juveniles studied. Our findings may indicate that the juveniles have greater survival chances in a suburban habitat. However, further studies are needed before such a conclusion can be confirmed.

The causes of death recorded in the study were often linked to anthropogenic effects such as shredding with garden waste, drowning in an artificial canal with high concrete edges, *Salmonella* infections transmitted at a feeding station, a possible dog attack and poisoning. This was an expected effect of cohabitation in a suburban environment.

### 4.3 Body mass change and nutritional status

Some individuals took a large effort to catch both before and after hibernation, causing their pre‐ and posthibernation body mass measures to be unrepresentative of the actual body mass loss during hibernation. It was possible to calculate the expected body mass on the date of hibernation onset for eight individuals, using regression lines. However, body mass gain after hibernation followed by a body mass loss for the males during the mating season made the body mass change appear polynomial instead, making calculations on expected posthibernation body mass measures uncertain. Comparing with previous studies of hibernation body mass loss in Danish, juvenile hedgehogs, Jensen (2004) calculated a mean body mass loss of 22.1% ± 10.1% (mean ± *SE*) during hibernation (*n* = 10), the lightest individual only losing 4.5% body mass (23 g). Rasmussen (2013) found that one individual went into hibernation weighing around 450 g and had only lost 20 g (4.5%) when retrieved in May 2013. Our estimate of mean body mass loss, calculated based on regression lines, was 16% ± 2.9% (mean ± *SE*) for individuals with an estimated prehibernation body mass of 379–1,149 g.

We used the Bunnell Index (Bunnell, 2002) as a supplementary measure of nutritional status. This index indicates the condition of an animal with regard to nutritional status, because body mass does not take skeletal size into account, and is therefore not necessarily a reliable measure of nutritional status. For example, a small hedgehog of 600 g would be in good condition, while a large hedgehog of 600 g would be in a poor condition. However, the results are completely dependent upon precise measures, which can be challenging in the field. Furthermore, the Bunnell Index also varies according the personality of the hedgehog, the degree of habituation to human contact and therefore the tendency for a hedgehog to curl up tightly or in a more relaxed manner. This proved a challenge especially when tracking body mass changes of specific individuals that would behave differently in the first, second and third weighing. Therefore, we found that the Bunnell Index was a somewhat unreliable method for determining nutritional status of hedgehogs. Based on the body mass change in the hedgehogs before and after hibernation, it appears that the individuals that could afford to lose most body mass did in fact lose most body mass, as was also seen in the study by Jensen (2004), Rasmussen (2013), and Morris and Warwick (1994).

### 4.4 Nests and nest changes during hibernation

Nest changes do take place during the hibernation period, and we radio tracked the hedgehogs on a weekly basis during the winter of 2014/2015. One individual changed nests eight times during hibernation and used seven different nests. However, it is possible that the individual did in fact change nests more frequently in between the events of radio tracking. Jensen (2004) found that the average number of nest changes during hibernation was two, with a single individual changing nests four times. Morris (1973) found that only two nests out of 167 studied remained occupied for the whole winter, and all others were vacated as the animals moved to another nest. Except for one individual, we found that the hedgehogs only changed nests one or three times, and several individuals did not change nests at all. However, if an individual had left the nest to forage and went back into the nest again, the activity would only have been recorded if the individual was out of the nest in the instant it was radio tracked. Nest change as a measure of activity during hibernation is somewhat difficult to detect and quantify accurately, which is why the use of accelerometers for future studies of hibernation would be a preferable method.

All nest types recorded were of good quality. They were situated under dense bushes, play houses, woodpiles, commercially manufactured hedgehog houses, and inside garden sheds. We observed a tendency for some individuals to place nests up against house walls, especially of older houses, for example, under woodpiles, in hedgehog houses situated on an undisturbed side of the house or even under permanent piles of bulky waste. This could be due to the radiation of heat from the lesser insulated houses.

### 4.5 The influence of climate

The Danish autumn and winter of 2014–2015 was exceptionally mild and wet. The autumn (September–November) was the second warmest since 1874 (average temperature of 11.9°C) and there were only 1.4 frost days in the period, which is exceptionally low compared with the long‐term average of 10 days. Autumn temperatures did not fall below freezing until the end of November. The winter was the ninth warmest since 1874, with an average temperature of 2.8°C and only 27.2 frost days. In addition, the seventh wettest winter since 1874 with a rainfall of 245 mm (December–February) in Copenhagen (Danish Meteorological Institute, 2015b). The mild autumn weather caused food resources like slugs and snails to be available until at least until the 1st of December, which is much later than normal. It is likely that these unusual weather conditions favorably influenced the survival of juvenile hedgehogs in this study.

It is well known that mild autumn weather influences the food availability, and hence the body mass change and survival as well as the breeding pattern of adult hedgehogs (Morris, 2018). Indeed, in our study we recorded two adult females giving birth to second litters during autumn 2014 (individuals 23–25 and 28 in this study, see Appendix S1), which is the first record of its kind in Denmark. Furthermore, many of the juveniles in this study entered hibernation much later than has been previously reported for Denmark (Jensen, 2004; Rasmussen, 2013; Walhovd, 1976, 1978, 1990), and we believe that this is likely linked to the exceptionally mild autumn weather.

Phenology studies of the UK hedgehogs have failed to detect an association between local weather conditions and the timings of posthibernation emergence of hedgehogs (PTES & BHPS, 2015). As found in previous studies (Jensen, 2004; Rasmussen, 2013; Walhovd, 1978), we observed that hedgehogs resumed activity after hibernation between mid‐April and mid‐May, after a winter with average climatic conditions (Danish Meteorological Institute, 2015b). However, our findings indicate that the start of hibernation may be influenced by local climatic conditions due to its effect on the availability of food resources. These results were subsequently confirmed by observations of delayed hibernation start and excellent body mass gain in Danish, juvenile hedgehogs during the mild autumns of 2015 and 2018 (Danish Animal Welfare Society, 2018; Danish Meteorological Institute, 2015a, 2018).

### 4.6 Radio tracking in a suburban habitat

Inferences from radio‐tracking data depend on the precision of the radio tracking. Fieldwork in this study took place almost every night in the autumn of 2014 and spring/summer of 2015. However, due to the wide distribution of study locations, we necessarily focussed on a single suburb per night, meaning that each individual was on average only followed one night per week. We therefore acknowledge that our tracking data likely do not represent the full extent of individual movement patterns and we may consistently underestimate home range size.

The suburban habitat of the hedgehogs proved to be a challenging environment for radio tracking. There was rarely visual contact whenever the hedgehogs moved around from garden to garden with high fences or hedges. Most location points were registered outside the actual garden in which the hedgehog would be present, making it impossible to observe their behavior. Constant access was granted to a number of gardens, especially the gardens with feeding stations, where most of the hedgehogs in the study were caught. The hedgehogs regularly visited the food bowls and this also turned out to be the easiest way to recapture the individuals for the weighing before and after hibernation.

### 4.7 Suggestions for future hedgehog studies

GPS tags can include built‐in radio tags and accelerometers and therefore contribute with more consistent data of spatial behavior and activity levels, providing information on the effect and importance of garden connectivity on hedgehogs and enabling researchers to obtain a larger sample size covering a larger geographical area, without disturbing and influencing the behavior of the hedgehogs studied (Barthel, Hofer, & Berger, 2019). Furthermore, the GPS tags could provide detailed information about the movement barriers affecting the hedgehogs in suburban areas. However, urban barriers may also in special cases deflect GPS signals.

Due to the intensified agriculture, with larger fields, the loss of hedgerows and grassland and use of pesticides, it is likely more beneficial to direct future conservation initiatives at the improvement and adaptation of urban and suburban habitats for hedgehogs to stop the drastic population decline seen in the UK (SoBH, 2011). It would therefore perhaps be more constructive to focus the research into hedgehog ecology on urban habitats, investigating which anthropogenic dangers that influence the survival of hedgehogs, how to reduce mortality rates and thereby target our conservation strategies toward hedgehogs in the most efficient way possible.

## 5 CONCLUSION

We radio tracked 35 juvenile hedgehogs in residential suburbs of western Copenhagen from 20 September, 2014 to 22 July, 2015. Nine individuals died, 12 lost their tags, and the signal was lost from seven individuals. The survival probability was high compared with previous research from Scandinavia. It is possible that the mild climatic conditions during the autumn of 2014 made food items abundant, causing the individuals to gain body mass fast and, in some instances, even delaying the onset of hibernation, which was considerably later than recorded in previous studies from Scandinavia. We furthermore recorded two incidences of second litters due to the favorable climatic conditions. Only two hedgehogs did not survive hibernation. Few hedgehogs seemed to lose more than 50 g during hibernation, and the heaviest hedgehogs lost most body mass. We found that home ranges became larger during the spring and summer of 2015 compared with the autumn of 2014, which was expected due to the onset of the mating season. The home ranges were generally smaller than those found in past studies from the UK, Norway, Sweden, and Denmark. However, previous research has not focused on juvenile hedgehogs in suburban areas.

Although our study has contributed with knowledge on the ecology of juvenile hedgehogs in suburban habitats and the potential impact of local climatic conditions on the behavior of the hedgehogs in the period of September 2014–May 2015 in a Danish setting, further studies representing more time series and locations are needed to provide the sufficient knowledge and data about hedgehog ecology in urban habitats to improve the conservation strategies in the area, and draw solid conclusions on the effects of climate change on hedgehog behavior.

## CONFLICT OF INTEREST

None declared.

## AUTHOR CONTRIBUTIONS

SLR conceived and led the project. SLR designed and conducted the fieldwork with advice from TD; SLR and ORJ analyzed the data with input from TBB; SLR wrote the manuscript with input from other authors. All authors gave final approval for publication.

## Data Availability

The remaining part of the dataset which is not represented in Appendix S1, is available for download from https://doi.org/10.5281/zenodo.3460362.
