# Supplementary material for: The ecology of suburban juvenile European hedgehogs (Erinaceus europaeus) in Denmark
Source: Ecol Evol. 2019 Oct 31;9(23):13174–87. doi: 10.1002/ece3.5764 (PMC6912878; doi:10.1002/ece3.5764)
Supplement: Supplementary file 1 [file ECE3-9-13174-s001.pdf]

Individual	Tagging date	Place	GPS location	♂/♀	Weight at tagging (g)	GPS coordinates, autumn 2014	GPS coordinates, spring/ summer 2015	Status July 2015	Cause of death	Nest changes	Nest types	Furthest distance autumn	Furthest distance spring/ summer	No. of gardens visited autumn	No. of gardens visited spring	Date of weighing	Weight (g)	BI	MCP50	MCP95	KDE50	KDE95
1	21/09/14	Taastrup	55.6401437<br>12.3246121	Female	213	32		Dead	Fox attack	0	Under a pile of leaves under a beech hedge behind a compost heap	78.5		7		02/11/2014	505	0.79	0.23	0.54	0.48	1.70
2	22/09/14	Taastrup	55.6396797<br>12.3235312	Male	213	30	11	Lost tag		1	Under a pile of leaves under a pine tree+ under garage shed (same garden)	133.4	156.3	7	7	29/10/2014<br>13/05/2015	570<br>653	0.81<br>0.74	0.06	0.38	0.24	1.15
3	24/09/14	Høje Taastrup	55.6424752<br>12.2713195	Female	348	12		Lost tag				550.8		3								
4	26/09/14	Taastrup	55.6379966<br>12.3230104	Female	562	31		Lost tag				317.6		11					0.90	2.25	2.57	8.76
5	26/09/14	Havdrup	55.5482930<br>12.1279890	Male	662	7		Lost tag														
6	26/09/14	Havdrup	55.5482830<br>12.1279110	Male	640	6		Lost tag														
7	27/09/14	Taastrup	55.6396703<br>12.3235160	Male	424	30	32	Alive		0	Under a densely growing bush	244.6	843.3	9	19	29/10/2014<br>14/05/2015<br>28/05/2014	755<br>665<br>692	0.86<br>0.80<br>0.79	0.06/<br>2.47	0.76/<br>14.61	0.45/<br>12.52	2.65/<br>48.75
8	27/09/14	Taastrup	55.6420365<br>12.3236088	Male	357	30	2	Signal lost		8	Under different piles of firewood, in piles of leaves under trees and bushes, in a garage, under pile of bulky waste	354.8		11		01/11/2014<br>24/04/2015	748<br>577	0.77<br>0.88	0.66	1.47	1.39	5.97
9	27/09/14	Taastrup	55.6420468<br>12.3236717	Female	359	15		Dead	Shredded with garden waste			907.9		9								
10	29/09/14	Høje Taastrup	55.6384470<br>12.2676425	Female	385	8		Dead	Fox attack			129		2								
11	1/10/14	Høje Taastrup	55.6359970<br>12.2783332	Male	342	7		Lost tag				399.1		5								
12	2/10/14	Rødovre	55.6967060<br>12.4474940	Male	301	5		Dead	Poison?													
13	4/10/14	Høje Taastrup	55.6412258<br>12.2672900	Male	396	6		Dead	Euthanasia, severe wounds and infections due to selfinflicted injuries caused by being stuck in a hawthorn bush													
14	5/10/14	Taastrup	55.6394241<br>12.3184153	Female	449	32		Dead	*Salmonella*	1	In compost heap and in hedgehog house (weighed at nest change)	188.3		13		16/11/2014	504	0.79	0.04	1.01	0.42	2.55
15	5/10/14	Taastrup	55.6381991<br>12.3170688	Female	450	16		Dead	*Salmonella*			216.5		10		02/11/2014	330	0.74				

Individual	Tagging date	Place	GPS location	♂/♀	Weight at tagging (g)	GPS coordinates, autumn 2014	GPS coordinates, spring/ summer 2015	Status July 2015	Cause of death	Nest changes	Nest types	Furthest distance autumn	Furthest distance spring/ summer	No. of gardens visited autumn	No. of gardens visited spring	Date of weighing	Weight (g)	BI	MCP50	MCP95	KDE50	KDE95
16	6/10/14	Brøndby Strand	55.6189752 12.4361155	Male	215	38		Lost tag		0	Under a pile of leaves under a privet hedge	210.5		11		03/11/2014 12/11/2014	476 543	0.82 0.86	0.51	1.40	1.00	3.70
17	20/10/14	Taastrup	55.6396910 12.3235770	Male	470	31	33	Alive		0	Under a garden shed	223.3	575.4	17	20	19/05/2015	754	0.71	0.56/ 2.84	1.64/ 4.51	1.12/ 5.53	4.40/ 20.22
18	20/10/14	Taastrup	55.6397620 12.3234830	Female	318	31	34	Alive		0	Under a pile of firewood in an open garage	83.4	272	5	13	16/11/2014 19/05/2015	480 645	0.79 0.86	0.18/ 0.18	0.36/ 2.13	0.30/ 0.77	1.04/ 5.08
19	19/10/14	Brøndby Strand	55.6196450 12.4358990	Male	318	6		Lost tag														
20	9/10/14	Vallens bæk	55.6491047 12.3623271	Male	385	30	2	Lost tag		3	In 3 neighbouring gardens, under beech hedges	241		20		11/11/2014	586	0.87	0.42	1.80	1.27	4.97
21	10/10/14	Islev	55.7046457 12.4494489	Male	190	6		Dead	Drowning in an artificial stream with concrete edges													
22	12/10/14	Alberts-lund	55.6645382 12.3336178	Male	659	2		Signal lost														
23*	15/10/14	Rødovre	55.6850130 12.4460750	Male	424	30	3	Dead	Dog/fox attack	3	Under pile of leaves under a beech hedge, in a compost heap	295.3		15					0.06	0.80	0.74	4.46
24*	15/10/14	Rødovre	55.6850620 12.4460230	Female	330	30		Lost tag				689.9		19					2.26	3.03	5.32	21.79
25*	19/10/14	Rødovre	55.6851150 12.4459890	Male	318	32	19	Lost tag		0	Under a play house	211.3	208.4	11	13	22/11/2014 21/04/2015	525 483	0.9 0.77	0.03	1.98	0.65	5.77
26	12/11/14	Rødovre	55.6789660 12.4611030	Female	330	3		Signal lost								12/11/2014	330	0.80				
27	30/11/14	Rødovre	55.6868984 12.4492683	Female	373	31	1	Lost tag (alive)		lost tag		205.9		16		30/10/2014	373	0.79	0.28	1.20	0.90	3.76
28	26/11/14	Rødovre	55.6789180 12.4614500	Female	256	10	6	Signal lost		0	In nestbox at a wildlife rehabilitation center	137.4		8		26/11/2014 25/05/2015 28/05/2015	256 551 531	0.85 0.85 0.89				
29	29/10/14	Taastrup	55.6394624 12.3181108	Male	488	31	10	Signal lost		1	In a bush near a house wall+ in a pile of leaves under a beech hedge	204.2	390.2	18	10	29/10/2014 19/05/2015	488 752	0.89 0.71	0.55	1.71	1.13	4.27
30	11/11/14	Høje Taastrup	55.6377470 12.2775210	Female	553	10		Lost tag		lost tag	In a densely growing bush surrounding a tree (ash)	366.2		6		11/11/2014	553	0.85				
31	17/11/14	Havdrup	55.5483220 12.1277680	Male	656	2	30	Alive		0	Under a pile of firewood in an open shed		324.9		11	02/11/2014 17/11/2014 17/05/2015	548 656 749	0.82 0.73 0.80	0.18	2.50	1.40	8.41
32	2/11/14	Havdrup	55.5483220 12.1277680	Female	589	2	30	Alive		1	Under a hen house+ in a dense bush alongside house wall (same garden)		218.4		13	02/11/2014 30/04/2015 10/06/2015	589 590 866	0.88 0.88 0.85	0.14	0.89	0.55	2.67
33	17/11/14	Havdrup	55.5483220 12.1277680	Male	822	1		Signal lost								02/11/2014 17/11/2014	541 822	0.89 0.81				
34¨	8/05/15	Rødovre	55.6850130 12.4460750	Male	681		30	Alive					417.1		19	08/05/2015	681	0.77	0.41	2.66	1.86	8.46
35¨	8/05/15	Rødovre	55.6850130 12.4460750	Male	577		31	Signal lost					476.3		19	08/05/2015	577	0.82	1.82	8.41	4.38	23.33
