# Supplementary material for: The ecology of suburban juvenile European hedgehogs (Erinaceus europaeus) in Denmark
Source: Ecol Evol. 2019 Oct 31;9(23):13174–87. doi: 10.1002/ece3.5764 (PMC6912878; doi:10.1002/ece3.5764)
Supplement: Supplementary file 2 [file ECE3-9-13174-s002.docx]

**Appendix S1. Overview of juvenile hedgehogs used in the study**

**Appendix S1.** A presentation of information on each individual used in the study. Two groups are siblings from the same litter (litters marked with * and ¨) and both groups have the same parents. Furthest distance is a measure of the distance between the two most distant location points registered per individual during the autumn of 2014 and spring/summer of 2015. 50MCP, 95MCP, 50KDE, and 95KDE are measures of home ranges in hectares. The black numbers indicate home ranges during the autumn of 2014, and the blue numbers represent the home ranges during spring/summer of 2015.
